# MicroRNA and Hemophilia-A Disease: Bioinformatics Prediction and Experimental Analysis

**DOI:** 10.22074/cellj.2021.7109

**Published:** 2021-07-17

**Authors:** Halimeh Rezaei, Majid Motovali-Bashi, Seyed Javad Mowla

**Affiliations:** 1 Department of Cell and Molecular Biology and Microbiology, Faculty of Biological Sciences and Technology, University of Isfahan, Isfahan, Iran; 2 Molecular Genetics Department, Faculty of Biological Sciences, Tarbiat Modares University, Tehran, Iran

**Keywords:** Bioinformatics, Factor VIII, HEK293 cells, Hemophilia-A, MicroRNAs

## Abstract

**Objective:**

Hemophilia-A is a common genetic abnormality resulted from decreased or lack of factor VIII (FVIII) pro-coagulant protein function caused by mutations in the *F8* gene. Majority of molecular studies consider screening of mutations and their relevant impacts on the quality and expression levels of FVIII. Interestingly, some of the functions in FVIII suggest a probable involvement of small non-coding RNAs embedded within the sequence of *F8* gene. Therefore, microRNAs which are encoded within the *F8* gene might have a role in hemophilia development. In this study, miRNAs production in the *F8* gene was investigated by bioinformatics prediction and experimental validation.

**Materials and Methods:**

In this experimental study that approved by Research Assistant, University of Isfahan, bioinformatics tools have been utilized to seek the novel microRNAs inserted within human *F8* gene. The ability to express new microRNAs in *F8* locus was studied through reliable bioinformatics databases such as SSCProfiler, RNA fold, miREval, miR-FIND, UCSC genome browser and miRBase. Then, expression and processing of the predicted microRNAs were examined based on bioinformatics methods, in the HEK293 cell lines.

**Results:**

We are unable to confirm existence of the considered mature microRNAs in the transfected cells.

**Conclusion:**

We hope that through changing experimental conditions, designing new primers or altering cell lines as well as the expression of vectors, exogenous and endogenous expressions of the predicted miRNA will be confirmed.

## Introduction

Hemophilia-A is a heterogeneous deficiency in blood coagulation factor VIII (FVIII), which causes increased bleeding and this occurs approximately 1 in almost 5000- 10000 male births. This involves problems originated from easy bruising as well as muscle and joint bleeds.

Almost, it is inherited as an X-linked recessive trait and classified into mild (>5% of normal level), moderate (1- 5% of normal level) or severe (<1% of normal level) on the basis of circulating levels of clotting FVIII. Plasma concentration of factor VIII is approximately 200 ng/ml and its biological half-life is nearly 12 hours (1).

FVIII is an essential plasma protein for blood coagulation that is bound to a von willebrand factor and circulates in the blood stream in an inactive form. FVIII is activated (FVIIIa) in response to injury and separates from von willebrand factor. Subsequently, activated FVIIIa interacts with FIX which is another coagulation factor. This interaction forms a blood clot with setting off a chain of additional chemical reactions. FVIII is encoded by the *F8* gene with 186 kb length located on Xq28 chromosome. It contains 26 exons and has two variant transcripts called "isoform a" and "isoform b". Many mutations are reported all over the *F8* gene. Inversions especially in the introns 1 and 22 are involved in 50% of total mutations and 1-4% of severe hemophilia-A patients, respectively. In addition, severe hemophilia-A have been reported from more than 120 large deletions (>50 bp) (1, 2). Other mutations in the *F8* gene include nonsense and missense mutations (point mutations), as well as small deletions and insertions that are diffused in all 26 exonic regions.

Up to now, about 1000 specific mutations with various types of origin have been collected from the global hemophilia database (HAMSTeRs) (3). In the past decade, non-coding RNAs (ncRNAs) were one of the most available biological findings.

microRNAs (miRNAs) are endogenous single-stranded ncRNAs with 18-25 nucleotides-long that mediate transcriptional and post-transcriptional control of the target gene expressions, as a part of complex gene regulatory networks and they are able to regulate some biological pathways (4). 

miRNAs can be found in various genomic regions including, introns of coding genes and 3ˊ un-translated region (3ˊ UTR) of coding genes (5), in addition to introns and exons of non-coding genes (6).

In most mammalian, RNA polymerase II transcribes miRNA genes (pri-miRNA), and their characteristics are the same as protein coding transcripts: a poly (A) tail, exons and a 5ˊ cap (7). The pri-miRNA, is rapidly trimmed into pre-miRNA precursor with about 70 nucleotides-long (8). The pre-miRNA is afterward transferred to the cytoplasm and was further accomplished to its mature shape, placed either at 5ˊ or 3ˊ side of the stem loop (9, 10).

In mammalian cells, mature miRNA mostly act via completing binding to 3ˊ-UTR of its target genes with its seed sequence. This leads to mRNA degradation or protein translation inhibition (11). To date, in miRBase database (http://www.mirbase.org/), more than 2000 human miRNAs have been published (12). In the human genome, approximately 55000 miRNA genes are expected to be present (13). Identification of novel miRNAs by numerous bioinformatics tools have been developed to be fast, effective and cheap (14, 15). 

A prosperous way to recognize miRNA genes in different plants and animals like human, mouse, Drosophila, C. elegans and others is using the bioinformatics approaches (16). The software is designed based on phylogenetic diversity and conservation, secondary structure information, thermodynamic parameters, stability of hairpin, sequence conservation in various species, sequence special parameters, similarity to the famous miRNAs and genomic position of the candidate sequences associated to the famous miRNAs (17- 19). Since hemophilia-A is a single-gene hereditary disorder, we decided to select *F8* gene for our genomic analysis. 

Here, in order to look for hairpin structures within the human *F8* gene, bioinformatics tools were utilized. 

The whole suitable bioinformatics characteristics for producing a real miRNA exist in two of the predicted stem loops. These conserved stem loops were experimentally investigated. In the present study, bioinformatics prediction and experimental validation for miRNAs detection in the F8 gene was analyzed.

## Materials and Methods

### Bioinformatics tools for prediction of miRNAs

RNA fold algorithm (http://rna.tbi.univie.ac.at/cgi-bin/ RNAfold.cgi), miREval (http://mimirna.centenary.org.au/ mireval/.) and SSC profiler programs (http://mirna.imbb. forth.gr/SSCprofiler.html) were employed to seek the probable hairpin structures in the area of interest. Target secondary structures dependent function of Drosha and Dicer enzymes, which have crucial roles in miRNAs biogenesis, inclined us to use the miR-FIND (http://140.120.14.132:8080/ MicroRNAProject-Web/). Conservation of the predicted miRNAs was examined using UCSC database (http://genome. ucsc.edu/), along with blat search for many organisms such as human genome. Furthermore, mature miRNAs in the candidate sequences were predicted by SSCprofiler. Using miRBase, similarity of our sequences was searched between 24,521 miRNAs loci from 206 species. This study approved by Research Assistant, University of Isfahan.

### DNA preparation

Hemophilia-A is a monogenic disorder and a defect in the *F8* gene resulting in this disease manifestation. On the other hand, most miRNAs directly affect and target their productive genes. Therefore, in order to predict the miRNAs involved in the control and regulation of hemophilia-A, *F8* gene was examined and analyzed only in healthy subjects.

Genomic DNA template was extracted from the person whole blood referring to Isfahan University Health Center (by receiving consent) according to Miller protocol (20). Based on bioinformatics studies, two candidate regions in *F8* gene were identified (we briefly refer them to can-miR-1 and can-miR-2, in this article).

These regions have ability to express the hairpin structure sequences belonging to possible miRNA precursors. Polymerase chain reaction (PCR) was performed with primers designed by Oligo v.7 and PerlPrimer 

(can-miR-1-

R: 5´-TTGTGGAGATTGAGTTCTGACC-3´

F: 5´-TAGAGACTCCCTTACGTGACTG-3´, 

can-miR-2-

R: 5´-AGCCTCCAAGGTGCTGTATAT-3´

F: 5´-CCTGCACTGAGCACTCATGAA-3´).

We used NCBI/Primer-BLAST to ensure that the primer sequences are unique. The thermocycler program for can-miR-1 consisted of one cycle for 5 minutes at 94˚C, 35 cycles for 30 seconds at 94˚C, 58˚C for 30 seconds and 72˚C for 30 seconds, followed by one cycle for 10 minutes at 72˚C, and for can-miR-2 it was designed by one cycle for 5 minutes at 94˚C, 35 cycles for 30 seconds at 94˚C, 62˚C for 30 seconds and 72˚C for 30 seconds, followed by one cycle for 10 minutes at 72˚C. Electrophoresis was performed in 1% agarose gel and the PCR products were analyzed in order to TA cloning (for double strand DNA); DNAs were purified and extracted using a GeNetBio Gel Extraction Kit (GeNetBio, Korea) (21). Segments into the TA vector pTZ57R/T (Thermo scientific, USA) were cloned into *Escherichia coli* strain *TOP10* based on standard protocol transformed (22). The transformed cells were then plated on LB agar improved with x-Gal (20 mg/ ml) and ampicillin (75 mg/ml). Colonies were accidentally chosen and DNA was utilized in colony PCR as templates. Positive colonies (transformed *Escherichia colis* trains) were verified in terms of the existence of inserts direction. Plasmid isolation was carried out using GeNetBio Plasmid Extraction Kit (GeNetBio, Korea) (21).

Recombinant TA vectors were digested by KpnI and SacI (at 37˚C for 10 minutes) restriction enzymes and they inserted into pEGFP-C1 expression vectors (cutting with the same restriction enzymes) that are downstream of the GFP gene and carrying CMV promoter and KpnI/ SacI restriction sites. Competent cells of *Escherichia coli DH5-α* were transformed by pEGFP-C1 contain DNA carrying pre-miRNAs and finally colony PCR was performed for transformation validation and inserts direction. Additionally, the hairpin structure sequence, as scrambled control (23), was cloned into the pEGFP-C1 vectors. As another negative control, the C1-mock (empty vector) was also utilized. For the reliability of correct inserts, all vectors were sequenced (Genfanavaran Co., Iran). 

### Cell lines

HEK293 was cultured in DMEM-HG including 100 U/ml penicillin, 100 µg/ml streptomycin and 10% fetal bovine serum (FBS) (all from Gibco, USA). After 24 hours culture in separate flasks, transfection was performed according to the calcium-phosphate protocol (24). In the first flasks, HEK293 was transfected with the main samples, which were recombinant expression vectors with insert fragments containing predicted can-miR-1 and can-miR-2 precursors. In the second flasks, HEK293 was transfected with pEGFP-C1- Scramble, which were recombinant expression vectors containing insert fragments (it should be noted that length of the insert fragments are approximately equal to the insert fragments in main vectors) and in the third flasks, HEK293 was transfected with pEGFP-C1 empty vectors (C1-Mock) to control potential effect of transfection reagent on the cell. In the last flasks, HEK293 was performed without transfection steps, as a control, using a florescence microscope. Finally, fluorescence microscopy was used to confirm the cells transfection and GFP expression.

### RNA extraction and preparation

Total RNA was extracted from HEK293 cell lines using Trizol reagent based on the manufacturer’s protocol (Sigma, Germany) (25) and treated with RNAase-free DNaseI (Takara, Japan) for 30 minutes at 37ºC followed by heat inactivation for 10 minutes at 65ºC by adding Ethylenediaminetetraacetic acid (EDTA).

Purity and quality of RNAs were estimated by NanoDropND-1000 (NanoDropTech; Thermo scientific, USA). In order to confirm the integrity of RNAs, 2% agarose gel electrophoresis were performed.

### Synthesis of cDNA

Universal cDNA Synthesis Kit II (Exiqon, USA) (26) was utilized for cDNA synthesis using OligodT primers and Reverse Transcriptase which have a universal tag sequence at the the 5′ end. Besides, polyadenylation of the mature miRNAs were applied for their reverse transcription. To confirm the expression of mature miRNAs, each cDNA sample was amplified using PCR.

Thermocycler program included one cycle for 5 minutes at 94˚C, 38 cycles at 94˚C for 30 seconds, 60˚C for 30 seconds and 72˚C for 30 seconds, followed by one cycle for 10 minutes at 72˚C. In addition, 13 and 14 primer sets for mature miRNAs of can-miR-1 and can-miR-2 were respectively designed according to bioinformatics analysis. They were used as forward primer and the primer in the buffer of cDNA Synthesis Kit was used as reverse primer. Then, to check PCR products, the samples were run on 12% polyacrylamide gel.

## Results

### Bioinformatics prediction of miRNAs

*F8* gene involves in hemophilia-A and it was observed by reliable bioinformatics databases due to elicit candidate stem-loops that express miRNAs. For this purpose, comprehensive studies were carried out on the related data servers. Regarding the achieved results from data servers, two stem-loop structures servers, two stem-loop structures can-miR-1 with sequence: (5´-CTCACCCTGACTTATC TGTTTCACAGAGTCCACATCTGGCCAATGGGAA ACACACCTTTTGCTCAGAAAGACCCTGGGAATG TAGGTCAATCATAATGCAGTAG-3´) and

can-miR-2 with sequence: (5´-CCTCACCCTCTTGCT GCTCAGCTCCAGGTCGTCGTGGGTTCAGGGCTC AGCTGCACGCTCCTGCCCGCGCCCTGGGCGTGA TGGCACCCCCAGCCCCTGCCATT-3´)

were eventually qualified and recommended for further experimental confirmations.

### Sequence, structure and conservation profiler web service

**Fig.1:**
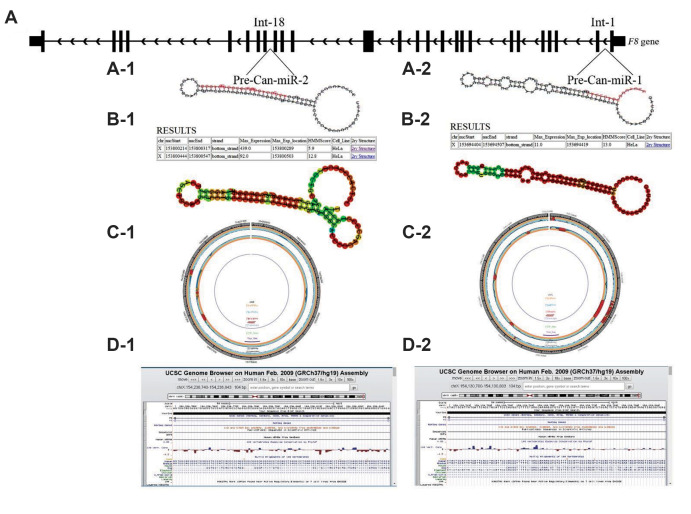
Prediction of pre-can-miR-1 and pre-can-miR-2 in the 1^st^ and 18^th ^introns of human *F8* gene, respectively. **A-1, A-2. **Results of SSC profiler for can-miR-1and can-miR-2. Hairpin structures containing a probable sequence of mature miR (Red) are shown, and HMM score related to the sestructures are shown in the tables. Furthermore, maximum expression (max-expression) according to a full genome tiling array in Hela cell line are presented for these sequences. **B-1, B-2. **Secondary structure results of can-miR-1 and can-miR-2 in RNA fold web server are depicted. **C-1, C-2. **About1000 bp are displayed around our inquiry sequences, as a circle graph by miREval. **D-1, D-2.** Conservation levels are shown with blue columns in UCSC genome browser.

This web service identifies stem-loop structures and it assigns a hidden Markov model (HMM) score apiece determined by applying conservation along with structure features (Fig.1A).

### RNA fold web server

The sequence of hairpin structures obtained from SSC profiler were imported to RNA fold web server for more detailed researches on their secondary structures and stabilities (Fig.1B). Calculated minimum free energies (MFE) for these structures are -26.80 Kcal/mol and -33.30 Kcal/mol, respectively.

### miREval

These sequences were also analyzed in miREval and the corresponding results are shown in Figure 1C.

### miR-FIND

Processing sites for Drosha and Dicer ribonuclease enzymes, mature miRNA sequences and the seed regions were determined for our mentioned sequences in this miRNA predictor service (Table 1).

### UCSC genome browser

Evolutionary conservation for the candidate sequences within 100 species, such as rhesus, mouse, dog, elephant and other vertebrates, were measured in the UCSC genome browser data server (Fig.1D).

**Table 1:** Analyzed information in miR-Find


Sequence	can-miR-1		can-miR-2	

Mature-miRNA Drosha/ Dicer processing site	23/46	76/56	15/45	83/62
Mature-miRNA sequence	5´-ACAGAGUCCAC AUCCGGCCAAUGG-3´	5´-CUUUUGCUCAG AAAGACCCUG-3´	5´-UGCUCAGCUCCAGGU CGUCGUGGGUUCAGGG-3´	5´-UGCCCGCGCCCU GGGCGUGAUG-3´
Predict seed site	5´-CAGAGUC-3´	5´-UUUUGCU-3´	5´-GCUCAGC-3´	5´-GCCCGCG-3´


Data corresponding to mature miRNA-5p and-3p are presented.

### MiRBase website

Ensuring non-registration within the previously identified miRNAs using miRBase was done for these sequences.

### DNA preparation

After genomic DNA extraction from whole blood, the concentrations and purity of isolated DNA samples were determined by NanoDrop and the samples were loaded on 1% agarose gel. 

The genomic regions containing a sequence of 104 nucleotides putative miRNA precursors were amplified by specific primers and PCR. In addition, by using100 bp DNA ladder, the bands were determined in the expected locations (370 bp and 679 bp, respectively).

The fragments containing predicted miRNA precursors were cloned into the TA vector. The transformation was carried out in the *TOP10* strains and then cultured in LB agar amended with ampicillin. Additionally, TA vector was cultured on another plate under the same conditions as a negative control sample.

In order to screen the positive colonies, they were randomly selected and colony PCR was performed using primers related to miRNA precursors and vector primers.

In spite of the existence of false bands, the presence of expected bands with ladder pattern confirmed the accuracy of direction of insertions.

In order to clone the fragments containing miRNA precursors in the expressed vector, isolated recombinant TA vectors were double digested by KpnI and SacI .The pEGFP-C1 expression vector by KpnI and SacI was double digested and then the products of this digestion were extracted from the gel.

The fragments containing miRNA precursor sequences were obtained by recombinant TA vector double digestion and inserted into the double digested pEGFP-C1 expression vector.

Then the insertion products transformed into *Escherichia coli* (*DH5-α* strains) and the strains plated on LB agar amended with kanamycin. Colony PCR was performed to select recombinant expression vector colonies containing insertion fragments in accuracy direction and gel electrophoresis confirmed this purpose. Then, plasmid isolation was performed. To verify isolation accuracy, the products were electrophoresed on the agarose gel.

**Fig.2:**
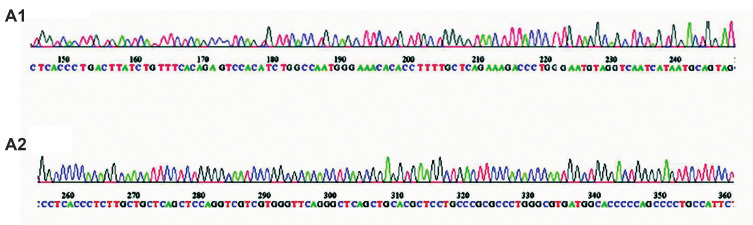
Sequencing results. **A-1, A-2. **When can-miR-1 and can-miR-2 sequences are cloned into the expression vectors must be in direction read the vectors sequence to be properly expressed, also no nucleotide changes were caused by the mutations in these sequences which do not produce defective RNAs. Outputs are confirmed accuracy of work.

Sequencing was carried out to approve sequence accuracy of the inserts inexpression vectors. Sequencing indicated 100% homology between the inserted sequence and the predicted miRNA precursor sequence (Fig.2).

### Cell lines

**Fig.3:**
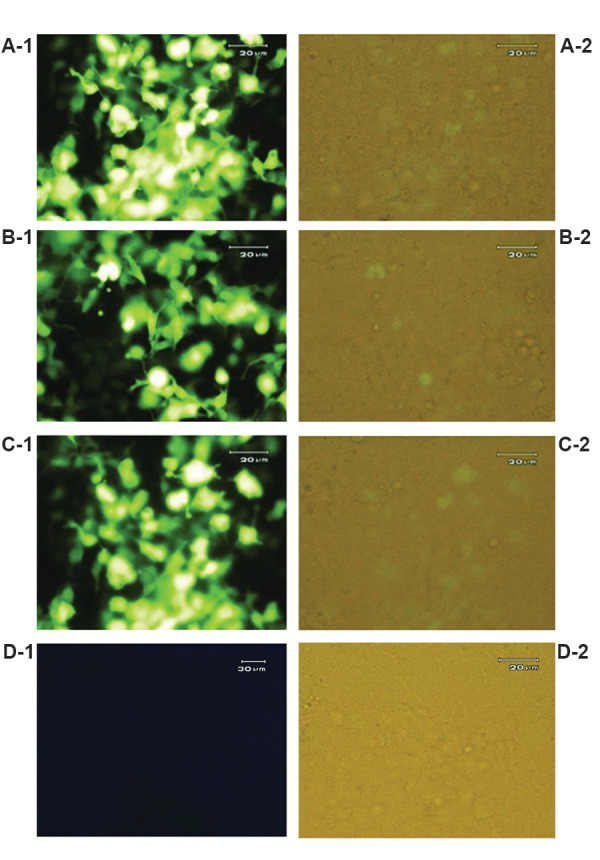
Observing GFP protein expression using florescence microscopy. GFP expression indicates transfection accuracy in **A-1, A-2. **Pre-miRNA, **B-1, B-2. **Scramble, **C-1, C-2.** Mock and **D-1, D-2.** Untransfected HEK 293 cell lines.

Transfection efficiency was evaluated by observing the cells during 36 hours after transfection, using florescence microscopy (Fig.3) for confirmation of recombinant plasmids expression in HEK293 cells.

### RNA extraction and preparation

About 48 hours after transfection, total RNA was extracted using Trizol. RNA concentration was determined using NanoDrop and RNA quality was assessed by loading samples on 2% agarose gel for confirmation of total RNA isolation accuracy.

### cDNA synthesis and examination of mature miRNAs expression

After cDNA synthesis, using PCR, whereby forward primers attached to the mature miRNAs and reverse primers attached to OligodT, the presence of mature miRNAs were studied. Due to insufficient information on the precise condition and sequences of candidate miRNAs, several forward primers were designed for the candidate miRNAs. These primers were designed according to the sequence recommended by the mentioned bioinformatics servers, for the mature miRNAs in the candidate precursor regions. After PCR, due to the small size of fragments and in order to better identification, PCR products were loaded on polyacrylamide gel. In addition, U6RNA was used as a reference gene. Due to the miRNAs length (about 22 nucleotides) and position of the universal primer on OligodT, 80-100bp fragments were expected.

**Fig.4:**
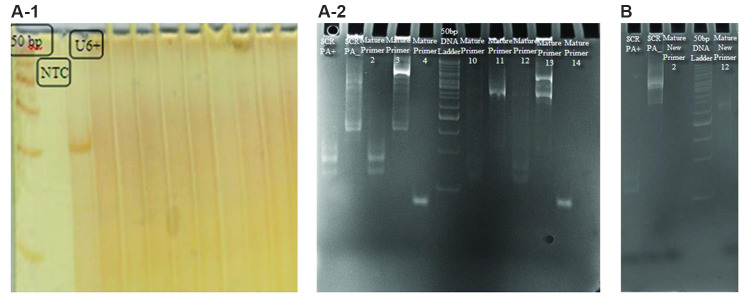
Polyacrylamide gel electrophoresis utilization to confirm the mature miRNAs expression. **A-1.** Wells related to negative control (NTC) and positive control (U6+) are indicated in the figure. The subsequent wells are related to polymerase chain reaction (PCR) products with primers designed for mature can-miR-1 at different annealing temperatures. **A-2.** Fragments for primers 2 and 12 with 80-100 bp were observed for can-miR-2 sequence. **B. **Two observed mature miRNAs of can-miR-2 with new sequence of primers loaded on polyacrylamide gel.

For can-miR-1, the expected band was not found in any of the designed primers (Fig.4.A-1). Two bands with 80-100 bp were observed for can-miR-2 (primers 2 and 12) in Figure 4A-2. Bands were prepared for sequencing after gel extraction. The sequencing results analysis confirmed existence of two predicted mature miRNAs, but there was not any additional nucleotide between the predicted can-miR-2 sequences and poly-A sequences. To approve the accuracy of mature can-miR-2 in comparison with the probability of error in replication by anchored oligodT, once again two forward primers for the observed sequences were designed by Oligo v.7 software and PerlPrimer (new sequence of primers 2 and 12 with sequences of 5´-GCTGCTCAGCTCCAGGTCG-3´ and 5´-TCAGCTGCACGCTCCTGC-3´, respectively) and PCR was performed. The samples were loaded on 12% polyacrylamide gel and no band was observed (Fig.4B).

## Discussion

Hemophilia-A is a result of a quantitative or qualitative defect in a plasma protein that is involved in blood clotting. It is a common genetic disease and one of the most serious hereditary blood disorders. So far, major molecular researches have focused on a variety of mutations in *F8* gene, particularly the effects of coding region mutation on the rate and quality of FVIII expression (27, 28). Researchers have found increasing evidences based on aberrant expressions of some miRNAs in various diseases by discovering miRNAs and proving their highly significant role in regulating expression of the most important genes.

While clarifying the molecular mechanisms involved in the relevant disorders, discovery of a miRNA associated with a disease helps meet diagnostic and therapeutic needs of the disease (29). So far, most of the miRNA identification has been accomplished by RNA cloning and sequencing. Various protocols have been developed for this purpose and have been successfully used to identify most of the current detected miRNAs.

All of these protocols follow the same rule, but there are differences in details of the method. The major limitation in identifying miRNAs with cloning is difficult to find miRNAs with low expression or in particular stages or limited cell lines. Another problem with these methods is problem to clone some miRNAs due to the physical characteristics, like sequence composition and posttranslational modifications (splicing, methylation). In addition to the above problems, cost and time-consuming will cause restrictions in these methods (30). In this regard, computational algorithms provide quick, efficient and inexpensive methods for predicting novel miRNAs in genomic sequences. Furthermore, predicting the genomic region of miRNA genes facilitates discovery of new genes by limiting the search to specific regions, but predicting the existence of miRNA genes by computational processes is not enough to prove these genes and the presence of predicted miRNA genes should be experimentally confirmed by examining the intrinsic expressions of the mature form of miRNA (31, 32). Although, more than 10 million transcribed loci would increase hairpin structures, not all of them are indeed cleaved to mature miRNAs in the human genome (33). Kim (32) indicated some conditions to mimic a small RNA as a miRNA: first, its expression should be confirmed by some methods like RT-PCR or primer analysis. Second, small RNA sequence should be located in a 60-80 nucleotides hairpin-structure, at either 5´ or 3´ arm of the hairpin without bulges or internal loops. Third, small RNA sequence should be conserved phylogenetically. Sequence conservation must be seen in the hairpin precursor (pri-miRNA) which is often less than mature miRNA. Finally, increase in miRNA precursor level by reducing Dicer function is a good witness for the existence of miRNA. However, due to problems and technical difficulties that exist in destroying Dicer in some cells, the latter criterion is not used similar to the other criteria.

Considering the conservation factor and the use of RNAfold database to analyze folding and minimum free energy, Yoon and Micheli. (34) predicted the structures of miRNA precursors and identified 38 novel human miRNAs among the structures that were highly similar to the identified miRNAs. Additionally, in the same fashion, Lai et al. (35) confirmed 24 novel miRNA genes by bioinformatics analysis.

By adopting a phylogenetic approach, Berezikov et al. (36) found that nucleotides in the stem sequence of miRNA precursor had significantly higher conservation than the other sequences in stem loop. Considering the matter as well as the other features of known miRNAs, they presented 69 potential candidates for the miRNA and confirmed expression of 16 mature miRNA by Northern blotting; thus 16 novel human miRNAs were identified. Furtheremore, by combining bioinformatics predictions with microarray analysis and direct cloning of sequences, Bentwich et al. (37) introduced 89 novel human miRNAs.

Using SSCProfiler, UCSC genome browser and several other databases, Dokanehiifard et al. (38) successfully predicted and validated two novel miRNAs in the *TRKC* gene and hsa-miR-6165 in *NGFR*. They also investigated their possible association with colorectal cancer. Additionally, they predicted and confirmed a new miRNA in *PIK3KCA* human gene with a possible role in colorectal cancer.

By developing a method based on miRDeep, Dokanehiifard et al. (39) discovered 99 putative novel miRNAs that were associated with neurodegenerative diseases. Saleh et al. (40) completely validated the novel hsa-miR-3675b inhibiting proliferation of human breast carcinoma cells. In this work, in order to identify and confirm candidate regions for the expression of miRNAs in *F8* gene bioinformatics methods were utilized. Two candidate regions with appropriate miRNA characteristics and the successful cloning of these areas were determined by the result of the bioinformatics studies. To express these miRNA precursors in human cells, recombinant human vector transfections were performed in a human cell line. It was expected that these cells would create their corresponding mature miRNAs using their processing system by expressing these precursors in the cells. Search for the mature miRNAs were carried out, but no result was achieved after RNA extraction and PCR using the possible mature miRNA primers. It is believed that expression of the predicted miRNA will be detected through altering cell lines and expression vectors or changing the experimental conditions. Another possible recommendation to confirm the candidate miRNA in the transfected cell lines includes PCR conditions, changing cDNA synthesis method or designing different primers. 

## Conclusion

Considering the findings obtained from this study, further studies are needed to confirm the presence of candidate miRNAs, in the light of the above points. After confirmation of the miRNAs at this stage, expression levels should be measured within the transfected and non-transfected cell populations using Real time-PCR. Since the protocols of this study were based on the published articles, bioinformatics prediction of miRNAs in different genes of HEK293 cell line has been experimentally validated. Moreover, expression of the mentioned miRNAs (can-miR-1 and can-miR-2) was confirmed by deep sequencing and RNA sequencing data. Confirmation of the presence of these miRNAs in the *F8* gene and their exogenous expression through the mentioned protocol is the primary goal of this research. We need real-time PCR technique to evaluate endogenous expression of the verified miRNAs. This will be the propsetive objective for future investigations. Changing type of the cell line and experimental conditions according to the recent protocols is considered to resolve the problems of experimentally confirmation of the candidate miRNAs in this article.

## References

[1] Salviato R, Belvini D, Radossi P, Tagariello G (2004). Factor VIII gene intron 1 inversion: lower than expected prevalence in Italian haemophiliac severe patients. Haemophilia.

[2] Lu M, Zhang Q, Deng M, Miao J, Guo Y, Gao W (2008). An analysis of human microRNA and disease associations. PLoS One.

[3] Kemball-Cook G, Tuddenham EG (1997). The factor VIII mutation database on the world wide web: the haemophilia a mutation, search, test and resource site HAMSTeRS update (version 3.0). Nucleic Acids Res.

[4] Aranha MM, Santos DM, Sola S, Steer CJ, Rodrigues CMP (2011). miR- 34a regulates mouse neural stem cell differentiation. PLoS One.

[5] Cai X, Hagedorn CH, Cullen BR (2004). Human microRNAs are processed from capped, polyadenylated transcripts that can also function as mRNAs. RNA.

[6] Rodriguez A, Griffiths-Jones S, Ashurst JL, Bradley A (2004). Identification of mammalian microRNA host genes and transcription units. Genome Res.

[7] Lee Y, Kim M, Han J, Yeom KH, Lee S, Baek SH (2004). MicroRNA genes are transcribed by RNA polymerase II. EMBO J.

[8] Ambros V (2004). The functions of animal microRNAs. Nature.

[9] Krol J, Loedige I, Filipowicz W (2010). The widespread regulation of microRNA biogenesis, function and decay. Nat Rev Genet.

[10] Wang Z (2010). MicroRNA: a matter of life or death. World J Biol Chem.

[11] Wang Y, Lee CG (2009). MicroRNA and cancer-focus on apoptosis. J Cell Mol Med.

[12] Griffiths JS, Grocock RJ, van DS, Bateman A, Enright AJ (2006). miRBase: microRNA sequences, targets and gene nomenclature. Nucleic Acids Res.

[13] Miranda KC, Huynh T, Tay Y, Ang YS, Tam WL (2006). A pattern-based method for the identification of MicroRNA binding sites and their corresponding heteroduplexes. Cell.

[14] Yoon BJ, Vaidyanat PP (2007). Computational identification and analysis of noncoding RNAs. IEEE Signal Processing Magazine.

[15] Oulas A, Reczko M, Poirazi P (2009). MicroRNAs and cancer-the search begins!. IEEE Trans Inf Technol Biomed.

[16] Lopez IdON, Schliep A, Carvalho ACPdLFd (2014). The discriminant power of RNA features for pre-miRNA recognition. BMC Bioinformatics.

[17] Berezikov E, Cuppen E, Plasterk RHA (2006). Approaches to microRNA discovery. Nat Genet.

[18] Berezikov E, Guryev V, van de Belt J, Wienholds E, Plasterk RHA, Cuppen E (2005). Phylogenetic shadowing and computational identification of human microRNA genes. Cell.

[19] Li L, Xu J, Yang D, Tan X, Wang H (2010). Computational approaches for microRNA studies: a review. Mamm Genome.

[20] Miller SA, Dykes DD, Polesky HF (1988). A simple salting out procedure for extracting DNA from human nucleated cells. Nucleic Acids Res.

[21] Available from: http://www.genetbio.com (22 Jun 2019).

[22] Hana H (1991). TOP10 E.coli competent cells (chemical transformation). Methods Enzymol.

[23] Xu N, Papagiannakopoulos T, Pan G, Thomson JA, Kosik KS (2009). MicroRNA-145 regulates OCT4, SOX2, and KLF4 and represses pluripotency in human embryonic stem cells. Cell.

[24] http://www.abcam.com/index.html?pageconfig=resource&rid=11471.

[25] https://www.sigmaaldrich.com/content/dam/sigma-aldrich/docs/Sigma/Bulletin/t9424bul.pdf.

[26] Redshaw N, Wilkes T, Whale A, Cowen S, Huggett J, Foy CA (2013). A comparison of miRNA isolation and RT-qPCR technologies and their effects on quantification accuracy and repeatability. BioTechniques.

[27] Friedman RC, Farh KKH, Burge CB, Bartel DP (2009). Most mammalian mRNAs are conserved targets of microRNAs. Genome Res.

[28] Kumar MS, Lu J, Mercer KL, Golub TR, Jacks T (2007). Impaired microRNA processing enhances cellular transformation and tumorigenesis. Nat Genet.

[29] Hogeweg P (2011). The roots of bioinformatics in theoretical biology. PLoS Comput Biol.

[30] John B, Enright AJ, Aravin A, Tuschl T, Sander C, Marks DS (2004). Human microRNA targets. PLoS Biol.

[31] Jima DD, Zhang J, Jacobs C, Richards KL, Dunphy CH, Choi WW (2010). Deep sequencing of the small RNA transcriptome of normal and malignant human B cells identifies hundreds of novel microRNAs. Blood.

[32] Kim VN (2005). MicroRNA biogenesis: coordinated cropping and dicing. Nat Rev Mol Cell Biol.

[33] Kang W, Friedlander MR (2015). Computational prediction of miRNA genes from small RNA sequencing data. Front Bioeng Biotechnol.

[34] Yoon S, Micheli GD (2006). Computational identification of microRNAs and their targets. Birth Defects Res C Embryo Today.

[35] Lai EC, Tomancak P, Williams RW, Rubin GM (2003). Computational identification of Drosophila microRNA genes. Genome Biol.

[36] Berezikov E, Guryev V, van de Belt J (2005). Phylogenetic shadowing and computational identification of human microRNA genes. Cell.

[37] Bentwich I, Avniel A, Karov Y, Aharonov R, Gilad S, Barad O (2005). Identification of hundreds of conserved and nonconserved human microRNAs. Nat Genet.

[38] Dokanehiifard S, Soltani BM, Parsi S, Hosseini F, Javan M, Mowla SJ (2015). Experimental verification of a conserved intronic microRNA located in the human TrkC gene with a cell type-dependent apoptotic function. Cell Mol Life Sci.

[39] Dokanehiifard S, Yasari A, Najafi H, Jafarzadeh M, Nikkhah M, Mowla SJ (2017). A novel microRNA located in the TrkC gene regulates the Wnt signaling pathway and is differentially expressed in colorectal cancer specimens. J Biol Chem.

[40] Saleh AJ, Soltani BM, Dokanehiifard S, Medlej A, Tavalaei M, Mowla SJ (2016). Experimental verification of a predicted novel microRNA located in human PIK3CA gene with a potential oncogenic function in colorectal cancer. Tumour Biol.

